# On-Table Extubation After Off-Pump Coronary Artery Bypass: A Step Forward to Fast-Track Recovery

**DOI:** 10.7759/cureus.86458

**Published:** 2025-06-20

**Authors:** Mohammed Aslam Hossain, Sanjoy Kumar Saha, Manish Mittal

**Affiliations:** 1 Cardiac Surgery, Bangladesh Medical University, Dhaka, BGD; 2 Cardiac Anesthesia, Bangladesh Medical University, Dhaka, BGD

**Keywords:** coronary artery bypass grafting, early extubation, erector spinae plane block, on-table extubation, postoperative recovery

## Abstract

On-table extubation following coronary artery bypass grafting (CABG) surgery has garnered attention owing to its potential to enhance postoperative recovery and reduce resource utilization. Traditional approaches often involve extended mechanical ventilation, which can delay discharge from the intensive care unit (ICU) and increase the risk of complications. This case report assessed the feasibility and outcomes of on-table extubation in a patient undergoing beating heart CABG.

A 42-year-old male with triple vessel disease underwent beating heart CABG under a combination of general and regional anaesthesia, including an erector spinae plane block (ESPB) at the T4 level for analgesia. Standard monitoring and anaesthetic protocols were adhered to, with careful titration of opioids and inotropes. Three grafts were placed: the left internal mammary artery (LIMA) to the left anterior descending artery (LAD), the reversed saphenous vein graft (RSVG) to the obtuse marginal artery 1 (OM1), and the RSVG to the posterior descending artery (PDA). Extubation was performed immediately after surgery based on the following predefined criteria: patient alertness, stable hemodynamics, and adequate oxygenation.

The patient was successfully extubated in the operating room with stable vital signs (blood pressure 110/70 mmHg, heart rate 96 beats per minute, SpO₂ >95%). Postoperative chest radiography and blood gas analysis revealed no abnormalities, and drain collection was minimal (300 mL on postoperative day 1). The patient was discharged on the eighth postoperative day without complications, demonstrating the safety and efficacy of on-table extubation.

This case supports the growing body of evidence that on-table extubation after CABG is safe and beneficial, reducing ICU stays and resource utilization without increasing the risks of reintubation or readmission. ESPB contributes to effective analgesia and hemodynamic stability, thereby facilitating early extubation. These findings are consistent with recent studies advocating early extubation protocols in cardiac surgery to improve recovery and patient outcomes.

## Introduction

In recent years, on-table extubation following coronary artery bypass grafting (CABG) surgery has gained significant attention. This approach has been recognised for its potential to enhance postoperative recovery and improve patient outcomes [1]. The technique involves the removal of the endotracheal tube, allowing the patient to commence independent breathing while still in the operating room immediately after the completion of the surgery. In contrast, traditional methods typically require postoperative mechanical ventilation until specific criteria are satisfied. This can prolong the stay in the intensive care unit (ICU) and delay the transition to outpatient care. We present a case of successful on-table extubation following beating heart coronary artery bypass surgery at Bangladesh Medical University (BMU).

## Case presentation

**Figure 1:**
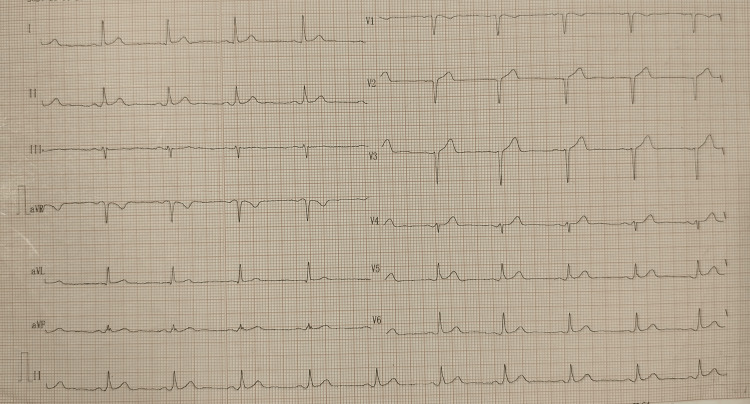
Electrocardiogram shows anteroseptal infarction with normal sinus rhythm.

**Figure 2:**
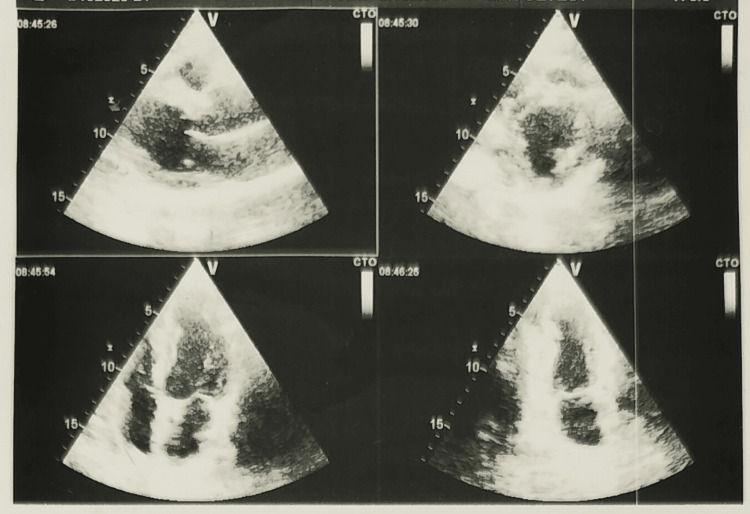
Echocardiogram shows that the distal mid-anteroseptal wall of the left ventricle is hypokinetic. Apical interventricular septum, mid apical anterior and inferior walls are hypokinetic. Mild to moderate left ventricular systolic dysfunction (ejection fraction 43%)

A 42-year-old male presented with cardiac complaints and was admitted to the Bangladesh Medical University. He had recurrent chest pain for six months - retrosternal, constricting, radiating to the left arm and shoulder, which was relieved by rest or sublingual nitroglycerin (GTN). Chest pain has recently been associated with mild exertion and daily activities. He experienced occasional breathlessness during exertion without diurnal variation. The patient denied orthopnea, post-nasal deep syndrome (PNDS), cough, syncope, leg swelling, or fever. He had hyperlipidaemia, a history of smoking, and a normal body mass index (23). He also had a history of myocardial infarction (MI), was treated with low-molecular-weight heparin (LMWH), and was discharged with a risk bond (DORB) from another hospital one month before admission to our hospital. On admission, pulse was 80/min, blood pressure 110/70 mm Hg, jugular venous pressure normal, respiratory rate 16/min, and temperature 98°F. Apex beat in the left fifth epigastric pulsation, normal first heart sound, single second sound, and clear lung base. The investigation showed that hemoglobin (Hb) was 11.4 g/dL, a normal biochemical marker. The patient's electrocardiogram shows normal sinus rhythm (Figure 1). The echocardiogram shows ejection fraction (EF) 43%, regional wall abnormalities, and good right ventricular (RV) function (Figure 2, Table 1). 

**Table 1:** Echocardiogram parameters IVSd: Interventricular Septum in Diastole, LVIDd: Left Ventricular Internal Diameter in Diastole, IVSs: Interventricular Septum in Systole, LVIDs: Left Ventricular Internal Diameter in Systole, EDV: End Diastolic Volume, ESV: End Systolic Volume, EF: Ejection Fraction, SV: Stroke Volume, LVd Mass: Left Ventricular Mass in Diastole

Parameters	Patient Value
IVSd	0.8 cm
LVIDd	5.1 cm
IVSs	1.1 cm
LVIDs	4.0 cm
EDV	124 ml
ESV	71 ml
EF	43%
SV	53 ml
LVd Mass	180.71 gm

**Figure 3:**
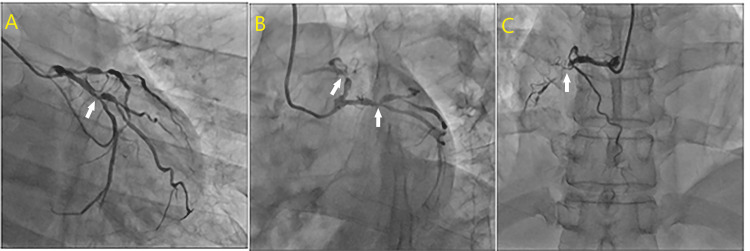
Both LAO (A) and RAO (B) views of the angiographic frame show an occlusion in the proximal or mid-segment of the left coronary artery, affecting the left anterior descending (LAD) and left circumflex (LCx) branches. The LAD appears faintly opacified with a visible cutoff, suggesting subtotal or total occlusion. Another RAO (C) view depicts the right coronary artery with a visible occlusion in the proximal segment. Contrast fails to fill the distal right coronary artery (RCA), confirming a complete or near-complete obstruction. LAO: left anterior oblique view, RAO: right anterior oblique view

Coronary angiography revealed an occluded proximal left anterior descending artery (LAD), 90% proximal left circumflex (LCx) stenosis, and a completely occluded right coronary artery (RCA). Impression was triple vessel disease requiring revascularisation (Figure 3). 

Written informed consent was obtained, and surgery was performed under combined general and regional anaesthesia. Before anaesthesia induction, an 18 G IV cannula was inserted with standard monitoring. The patient was preoxygenated and subsequently induced with 100 mg of thiopental, 50 mcg of fentanyl, isoflurane, and 6 mg of vecuronium, followed by intubation using an 8.0 endotracheal tube. Following intubation, a 20 G left radial intra-arterial cannula, a right internal jugular vein central venous cannula, a 14 F nasogastric tube, and an oesophageal temperature probe were inserted. In the left lateral position under aseptic conditions, a bilateral erector spinae plane block (ESPB) was performed at the T4 level using ultrasound. The probe was positioned longitudinally over the vertebral spine and subsequently moved laterally until the transverse process and paraspinal muscles were identified. The needle was then inserted from the cephalic side of the probe until it reached the transverse process. After negative aspiration, a local anaesthetic mixture (25 ml plain bupivacaine 0.2% and 50 mcg dexmedetomidine) was injected with ultrasonographic visualisation. The patient was then placed in a supine position for surgery. Anaesthesia was maintained using isoflurane and fentanyl. Fluid loading, GTN, and noradrenaline were titrated to maintain stable haemodynamics.

Three grafts were placed on the beating heart: the left internal mammary artery (LIMA) to the LAD, reversed saphenous vein graft (RSVG) to the obtuse marginal artery 1 (OM1), and RSVG to the posterior descending artery (PDA). The procedure was performed without any complications. No transfusion was used as blood loss was minimal, approximately 200 ml, replaced with crystalloids, and intake output was balanced. Following surgical haemostasis and sternal closure, anaesthetic drug delivery was stopped. After skin closure, extubation occurred once the patient was alert and responsive and maintained stable haemodynamics with minimal inotropes (noradrenaline 0.04 mcg/kg/min). During extubation, blood pressure was 110/70 mmHg, heart rate 96 b/min, temperature 98.0°F, and O₂ saturation >95% with 6 L/min oxygen via a face mask, with normal ECG rhythm; however, the patient reported slight discomfort. Postoperative analgesia was prescribed as injectable paracetamol (1 g every eight hours), along with 20 mg of nalbuphine, as required. Post-intensive care unit chest radiography and blood gas analysis revealed no abnormalities. The drain collection volume was 300 ml on the first postoperative day. The patient was transferred to the ward on the second postoperative day and was discharged from the hospital on the eighth postoperative day, with good stability.

## Discussion

As our patient fulfilled the extubation criteria [2] in the operating room, an on-table extubation was performed. CABG typically requires a longer cardiopulmonary bypass (CPB), leading to extended mechanical ventilation and ICU admission [3,4]. High doses of opioids during cardiac surgery necessitate prolonged postoperative mechanical ventilation. In our patient, 350 µg of fentanyl was administered during surgery. Delayed extubation is mainly for individuals at high risk of cardiorespiratory complications [5]; our patient had no such complications postoperatively. A systematic review and meta-analysis showed that the incidence of prolonged mechanical ventilation (PMV) among patients undergoing cardiac surgery is approximately 20%. This finding confirmed that many patients required prolonged postoperative ventilator support. The review highlighted that PMV is associated with increased in-hospital mortality. The odds ratio for mortality in patients requiring PMV was significantly high, indicating that prolonged ventilation is a critical risk factor for adverse outcomes [6].

Studies have shown that on-table extubation leads to a shorter ICU stay (32 hours vs. 39 hours), hospital stay, and lower resource utilisation. As in our first case, we discharged the patient on the eighth postoperative day to observe any complications. On-table extubation did not compromise the patient's safety. The rates of reintubation, ICU readmission, and 30-day readmission rates were similar between patients extubated on and later [7]. Similarly, Jaquet et al. (2023) found that on-table extubation was associated with a lower postoperative pneumonia risk and fewer vasopressor requirements [8].

Studies have shown that patients experiencing PMV face complications, including respiratory failure and pneumonia, and have significantly longer ICU stays and higher complication rates than those extubated earlier [9]. The long-term outcomes in patients requiring PMV are concerning. Research indicates that those weaned from mechanical ventilation after prolonged periods have poorer quality of life and increased morbidity [10-12].

Prolonged mechanical ventilation hampers neurocognitive recovery and demands nutritional support to prevent catabolic states following cardiac surgery [13]. In our case, we demonstrated that combined ESPB with simple analgesia may provide adequate analgesic haemodynamic stability for off-pump coronary artery bypass surgery, which can facilitate on-table extubation.

## Conclusions

On-table extubation following CABG can be a safe practice that improves recovery time and reduces resource utilisation without compromising patient safety. As evidence supporting this practice grows, surgical teams must adopt appropriate protocols and selection criteria to maximise the benefits. This case highlights the potential for early extubation in cardiac surgery, reaffirming its value in improving patient care and recovery.
